# Reverse Koebner Phenomenon in a Vitiligo Patient Treated With Radiotherapy

**DOI:** 10.7759/cureus.60771

**Published:** 2024-05-21

**Authors:** Asma S Khan, Quratulain Badar, Kaynat Siddiqui, Shoaib Hanif, Hafeez Lakhan

**Affiliations:** 1 Department of Radiation Oncology, Dr. Ziauddin Hospital, Karachi, PAK

**Keywords:** radiotherapy, reverse koebnerization, koebner phenomenon, re-pigmentation, vitiligo

## Abstract

Radiation-induced hypopigmentation resulting in a skin condition similar to vitiligo is evident in limited studies. In contrast to the typical Koebner phenomenon where new lesions develop at the site of injury, the trauma-induced disappearance of a specific rash in a patient with an already-developed skin disease is seen very rarely. This phenomenon is called "reverse Koebnerization" or "Koebner non-reaction." Herein, we submit a case of a 51-year-old female with already-developed vitiligo who came for treatment for carcinoma of the tongue with radiation therapy. Later, after the treatment, the patient developed a re-pigmentation of her skin.

## Introduction

Vitiligo is a skin disorder that is characterized by patches of depigmented skin that are white and well-defined [1]. Although the exact etiology is unknown, the primary cause of this chronic skin disorder is thought to be an autoimmune-mediated cell death of melanocytes and a loss of function of melanocytes that results in decreased melanin production in the epidermis of skin and hair follicles [1]. The molecular mechanisms are not yet fully determined [2].

“Koebner phenomenon” refers to a condition where new lesions of a particular disease appear after trauma to an area of skin that was previously unaffected by the disease [3-6]. This phenomenon was particularly observed in cases of psoriasis, vitiligo, and lichen planus. The type of trauma can be a physical injury, chemical injury, thermal injury, an allergic reaction, ultraviolet light, or ionizing radiation [7]. The reverse Koebner phenomenon is very scarce. There are only a few cases reported in the literature. Cases of the reverse Koebner phenomenon after exposure to ionizing radiation are even fewer. In 2013, a case series of the reverse Koebner phenomenon was published, in which they reported only four cases of radiation-induced reverse Koebnerization in the literature review [8]. Herein, we report this very unusual occurrence of reverse Koebnerization in a 51-year-old female patient with generalized vitiligo who was treated for carcinoma of buccal mucosa with radiation therapy as part of her treatment regimen.

## Case presentation

**Figure 1:**
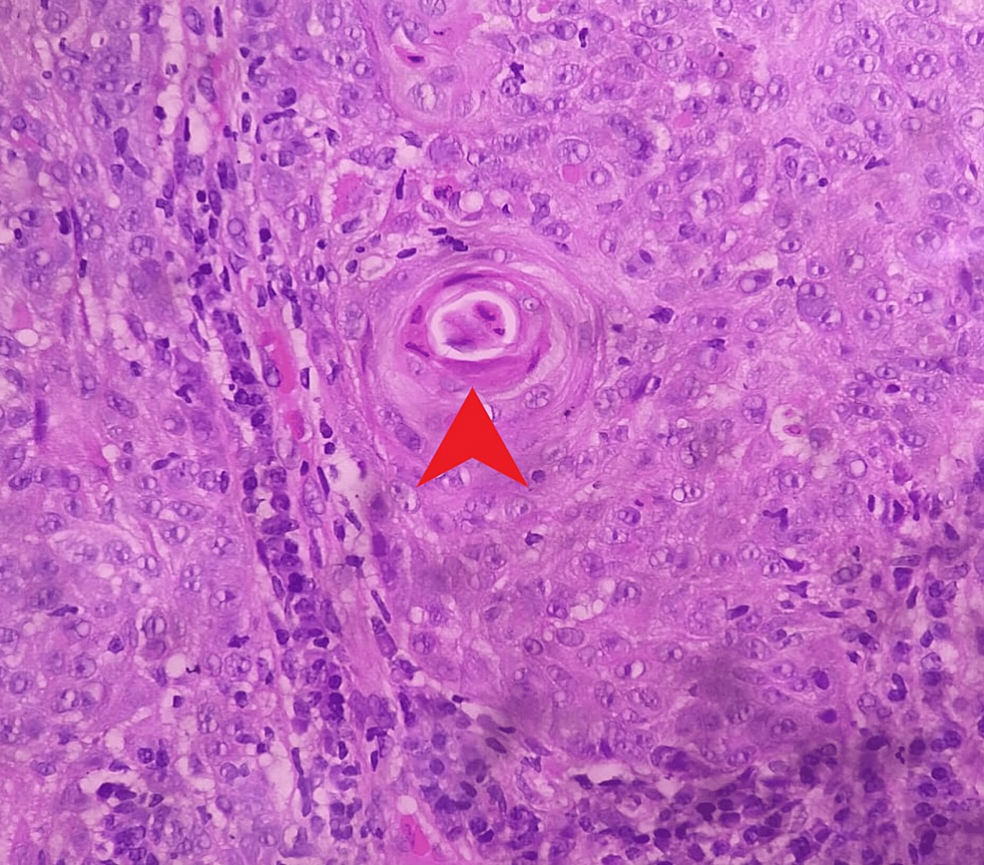
Biopsy-proven moderately differentiated squamous cell carcinoma with a red arrowhead showing keratin pearl formation

A 51-year-old lady with generalized vitiligo was diagnosed with moderately differentiated squamous cell carcinoma (Figure 1) of the left lateral border of the tongue in July 2022. She underwent a partial glossectomy with a radical left neck dissection. Histopathology showed a moderately differentiated squamous cell carcinoma. The disease was staged as IVA according to the tumor-node-metastasis (TNM) staging system.

**Figure 2:**
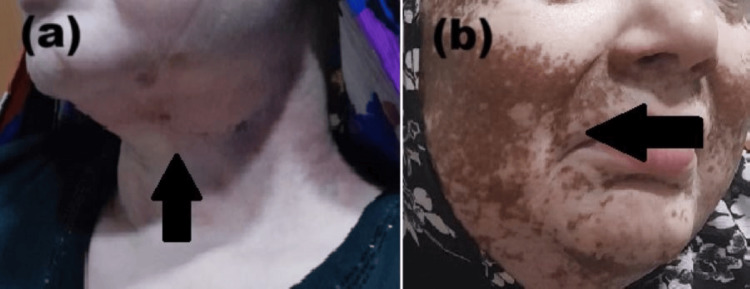
Post-treatment follow-up pictures (a) On a three-month-follow-up (arrow) showing the initiation of re-pigmentation over the neck (b) On a six-month post-treatment follow-up (arrow) showing marked re-pigmentation over the face

After the surgery, concurrent chemoradiotherapy was planned as an adjuvant treatment as per the National Comprehensive Cancer Network (NCCN) guidelines. She received cisplatin 40mg/m^2^ weekly dose for six cycles concurrently with radiation therapy with 6MV photon energy. The maximum tissue absorbed dose of 66Gy in 33 fractions to the oral cavity and 54Gy in 27 fractions to the bilateral neck by the intensity modulated radiation therapy (IMRT) technique over nine weeks total elapsed days 53. During her treatment, she developed grade IV mucositis along with grade II dermatitis which was managed conservatively. Dermatitis and mucositis resolved after six weeks of treatment. Post-radiotherapy treatment, she was kept on follow-up every three months. At three months of follow-up, to our surprise, her skin started to develop a few areas of re-pigmentation at the irradiated neck region (Figure 2a). The patient was advised to continue the skin protectant ointment with panthenol and glycerin, 1% silver sulfadiazine, and 1% hydrocortisone cream. However, at the six-month follow-up, the skin over the face and neck of the irradiated region showed marked re-pigmentation which started from the neck and then spread over the cheeks. (Figure 2b). The patient was referred to a dermatologist who confirmed the re-pigmentation of the skin affected by vitiligo. She was advised to continue using the 1% hydrocortisone cream and ultraviolet sunblock; no specific treatment was recommended for the re-pigmented skin. The patient was advised for a six-month follow-up.

## Discussion

Vitiligo is a chronic disease that results in depigmentation of the skin, leading to patches of white skin that vary in size and location. The etiology behind it is not well understood but a combination of factors that include genetic, autoimmune, and environmental are thought to play their role [1,2]. Heinrich Koebner described the Koebner phenomenon for the first time in a psoriasis patient. It refers to the appearance of new lesions at the site of local trauma on the previously uninvolved skin in an already diseased person. The phenomenon is not limited to one type of trauma and can be reproducible. Koebner phenomenon produced in the exposed area without any widespread lesions induced by ionizing radiation therapy has been reported in the medical literature [9]. “Reverse Koebner phenomenon”, also known as the inverse Koebnerization or isomorphic nonreaction, is a unique and understudied occurrence in which the existing lesions of a disease regress and sometimes completely vanish at the site of trauma [3]. Rather than triggering the development of new lesions, trauma stimulates the healing of the existing ones [5].

Medical literature has reported very few cases of this phenomenon associated with radiation therapy. Reverse Koebnerization is seen in a patient with psoriasis treated with ionizing radiation [4]. The literature lacks any report on reverse Koebnerization in a vitiligo patient treated with ionizing radiation. Kannangara et al. published a case series on the reverse Koebnerization of their case series of reverse Koebner phenomenon; according to their literature review, 16 cases have been reported to date, and only four cases are of radiation-induced reverse Koebnerization [8]. Cochran and Wilkin in 1981, reported a case of reverse Koebnerization in a 12-year-old girl who was irradiated for Wilms tumor at the age of six years, six years later she developed a drug-induced rash which surprisingly spared the irradiated field [10]. Bernhard and Haynes in 1982, reported a similar case of a 26-year-old female who received radiation to the liver and after a few days developed this phenomenon [5]. Nasca et al. in 1994, shared a case of this sparing phenomenon in a patient treated with radiation for lung adenocarcinoma and later developed a steroid acne sparing the irradiated scapular region [11]. Martin et al. in 2006, reported a very similar case to our case, of reverse Koebnerization in a female with psoriasis treated with radiation therapy for the treatment of carcinoma of the breast [4].

The cause and mechanisms underlying it remain obscure. It is anticipated that the immune system, inflammatory responses, and wound-healing processes may interact in complex ways to produce this phenomenon [12]. This could be due to cellular alterations like loss of Langerhans cells in the skin due to irradiation, alterations in the vascular wall due to irradiation resulting in reduced activity of the blood vessel wall, and radiation-induced cytokine imbalance causing prevention of overexpression of the type-1 proinflammatory cytokines which are thought to be responsible for development and maintenance of psoriatic lesions [4,5,11].

Further research is required to elucidate the mechanisms involved to explore potential therapeutic implications for individuals with skin conditions affected by this phenomenon.

## Conclusions

The reverse Koebner phenomenon is an uncustomary dermatological condition, the appearance of which is not restricted to a single type of insult. It was reported for the first time in a patient with psoriasis; later, the condition was seen in a few patients with vitiligo. In some cases treated with ionizing radiation, prevention of the development of new skin lesions in the irradiated field was seen due to the absence of cutaneous immune reactions. Whereas, the disappearance of the already-developed lesions was seen in a few. The pathogenesis of the underlying condition is obscure due to its unique presentation. Some authors have suggested that this may be due to a cytokine imbalance caused by the reduced expression of cytokines, which are typically responsible for initiating, maintaining, and recurring skin lesions, or due to some vascular factors such as nonreactivity of the blood vessels and decreased vascular bed. The exact cause is still undetermined, and further research is needed.
